# Clinical Thresholds for Visceral Adiposity Accumulation: A Comparative Analysis in Sex‐, Age‐, and BMI‐Matched Black and White Adults

**DOI:** 10.1002/ajhb.70165

**Published:** 2025-11-05

**Authors:** Austin J. Graybeal, Nuno Oliveira, Molly F. Johnson, Maria G. Kaylor, Abby T. Compton, Sydney H. Swafford, Caleb F. Brandner, Jon Stavres

**Affiliations:** ^1^ Department of Kinesiology & Nutrition, Harris College of Nursing and Health Sciences Texas Christian University Fort Worth Texas USA; ^2^ School of Kinesiology & Nutrition, College of Education and Human Sciences University of Southern Mississippi Hattiesburg Mississippi USA; ^3^ Department of Health and Human Physiology, College of Liberal Arts and Sciences University of Iowa Iowa City Iowa USA; ^4^ Department of Kinesiology, Nutrition, and Health, College of Education, Health, and Society Miami University Oxford Ohio USA

**Keywords:** anthropometrics, body composition, health disparities, obesity, visceral adipose tissue

## Abstract

**Objectives:**

This study aimed to prospectively identify visceral adipose tissue (VAT) accumulation thresholds in a cohort of sex‐, age‐, and BMI‐matched non‐Hispanic White and Black adults using a range of commonly employed whole‐body and abdominal‐specific adiposity measures associated with chronic disease risk.

**Methods:**

A total of 344 non‐Hispanic White (*n* = 172) and Black adults (*n* = 172) matched for sex, age, and BMI completed anthropometric and DXA‐based body composition assessments. Anthropometric measures included BMI, waist circumference, waist‐to‐hip ratio (WHR), and waist‐to‐height ratio (WHtR). DXA was used to quantify VAT, subcutaneous adipose tissue (SAT), body fat percentage (BF%) across the whole body, trunk, and android region, and the android‐to‐gynoid BF% ratio. Segmented linear regression was used to identify significant sex‐ and race‐specific VAT thresholds for each variable—defined as the inflection points where the relationship between VAT and each variable significantly changed.

**Results:**

Significant VAT thresholds were observed for BF%, WHtR, SAT, android BF%, and trunk BF% in both racial groups, with all thresholds higher for Black than White adults. When stratified by sex, all variables showed significant thresholds in White males, while none were observed in Black males. Significant VAT thresholds were identified for BF%, WHR, and SAT in Black females; WHtR in White females; and android‐to‐gynoid BF% in both groups.

**Conclusions:**

After matching for key anthropometric influences, distinct sex‐ and race‐specific VAT thresholds exist for Black and White adults, often falling below current clinical guidelines. These findings support the need for population‐specific screening tools to improve early detection and prevention of cardiometabolic risks.

## 1 Introduction

Abdominal obesity is associated with an increased risk for systemic inflammation, hypertension, type II diabetes (T2D), cardiovascular disease (CVD), and mortality (Chait and den Hartigh 2020; Price et al. 2006). While abdominal obesity itself presents an increased risk for several metabolic abnormalities, conventional models of abdominal adiposity demonstrate that specific body fat (BF) distribution patterns, which vary by sex, differentially impact disease. For example, during periods of increased adiposity, males are more likely to develop the more pathogenic visceral adipose tissue (VAT), whereas females tend to preferentially store fat in the subcutaneous adipose tissue (SAT) depots (Nauli and Matin 2019). In fact, previous studies have demonstrated that greater VAT is associated with worsening cardiometabolic health parameters (Bosch et al. 2015; Chait and den Hartigh 2020), with VAT accumulation occurring at substantially lower whole‐body fat percentages (BF%) in males (Bosch et al. 2015; Watson et al. 2019).

VAT accumulation and its impact on cardiometabolic health also varies across racial and ethnic groups (Wells 2009, 2012). For instance, non‐Hispanic Black adults consistently exhibit lower levels of VAT compared to non‐Hispanic White adults (Albu et al. 1997; Camhi et al. 2011; Katzmarzyk et al. 2010), yet paradoxically demonstrate the highest prevalence of obesity, CVD, and diabetes (Borkowski et al. 2024; Hales et al. 2020; Mensah 2018). Notably, Black women have experienced the greatest increase in metabolic syndrome prevalence, a trend largely attributed to increases in abdominal obesity (Gaillard 2018), despite typically having less VAT than their White counterparts. These observations suggest that the well‐established associations between VAT, overall adiposity, and cardiometabolic health (Rothney et al. 2013) may be moderated by ethno‐genetic heritage.

Often conflated with race, which is more accurately understood as a social construct than a biological one (Chaturvedi et al. 2025; Duggan et al. 2020), the concept of ethno‐genetic heritage serves as a moderating factor that encompasses ethnicity (defined by traditions, diet, and other lifestyle behaviors), genetic ancestry (referring to biologically relevant genetic markers), and, where applicable, racial identity, acknowledging that race reflects socially mediated experiences that often converge with biologically inherited traits. For example, individuals with isolated hypertension are more likely to be Black and have lower levels of education, income, and health insurance coverage (i.e., social constructs) (Graybeal, Compton, Swafford, et al. 2024; Grebla et al. 2010) coinciding with inherited and epigenetically influenced mechanisms of hypertension for this group, such as higher salt‐sensitive blood pressure reactivity (Elijovich et al. 2024) and epigenetic regulation of cardiac gene expression via promoter methylation (Pepin et al. 2021). This convergence is supported by studies showing that race correlates with perceived ancestry (Banda et al. 2015; Oni‐Orisan et al. 2021) and other phenotypic traits (e.g., race coinciding with observable/measurable adipose tissue distribution patterns that are the result of an interaction between one's genotype and their environment (Albu et al. 1997; Camhi et al. 2011; Katzmarzyk et al. 2010)) that contribute to disease development. It is for this reason that race, while socially constructed, has long been used as a surrogate for both genetic ancestry and social determinants of health, as it reflects the intertwining influence of biological and social constructs. Stress, as well as other socioeconomic factors such as limited greenspace, access to healthy food, reduced educational opportunities, and decreased physical activity disproportionately affect Black individuals (though White individuals may also experience these to a lesser extent) and are associated with increased VAT (Chagas et al. 2025; Lee et al. 2017), yet other studies indicate that discrimination, including racism, is similarly linked to VAT accumulation in both White and Black females (Lewis et al. 2011). While these social health determinants may explain a VAT‐independent mechanism of cardiometabolic disease development for Black adults (i.e., the aforementioned paradoxical relationship), they do not sufficiently identify the differential VAT accumulation patterns consistently observed between race groups. Together, these findings suggest a more complex interplay of social and biological influences embedded within the ethno‐genetic differences in VAT for White and Black adults. Accordingly, more evidence is needed to clarify patterns of VAT accumulation among White and Black males and females, as well as to establish healthy ranges for these patterns.

It is postulated in some models that excessive fat accumulation imposes mechanical strain on subcutaneous adipocytes, limiting their capacity for expansion and resulting in the rapid redirection of circulating lipids into visceral depots—a phenomenon commonly referred to as ‘the spillover effect’ (Virtue and Vidal‐Puig 2010). Highlighted in overfeeding studies (Alligier et al. 2013), this theory proposes that the relationship between VAT and whole‐body fat accumulation is non‐linear (Bosch et al. 2015; Kelly et al. 2020), with VAT accumulation occurring at specific ‘thresholds’ during increases in total adiposity, defined as the inflection point where the slope of the otherwise linear relationship changes. Interestingly, studies have reported no differences in VAT between Black and White adults after correcting for total BF (Hill et al. 1999). Moreover, VAT accumulation thresholds appear to be lower for males and White individuals (Bosch et al. 2015; Kelly et al. 2020), which may help explain the consistently lower levels of VAT observed in females and Black adults. However, to our knowledge, no studies have identified the differences in VAT accumulation thresholds for White and Black males and females using commonly applied clinical and research‐grade measurements of whole‐body and abdominal‐specific composition, with existing studies being limited to youth and adolescent populations (Kelly et al. 2020).

Given that accurate assessments of VAT are often inaccessible and rarely utilized in clinical practice (Graybeal, Brandner, Tinsley, et al. 2023), there have been growing calls to establish sex‐ and race‐specific thresholds using whole‐body assessment techniques (Liu et al. 2024); with these calls beginning almost two decades ago (Carroll et al. 2008). Identifying these thresholds could not only help clarify potential differences in health outcomes between Black and White adults, but also support efforts to address inequities in these outcomes. Such insights, when combined with assessing individual‐level factors to avoid stereotyping, could also improve clinicians' ability to more effectively screen for cardiometabolic risk in Black adults and help reduce the underdiagnosis and misclassification that contributes to persistent racial health disparities. Moreover, inherent differences in anthropometric characteristics between White and Black adults influence BF distribution patterns (Katzmarzyk et al. 2010). While it is common to account for these differences by including relevant characteristics as covariates in linear regression models (Bosch et al. 2015; Watson et al. 2019), excessive covariate inclusion can lead to model overfitting, ultimately reducing the ability to replicate findings in external populations (Zhang 2014). Additionally, this approach assumes linearity, which may not hold when non‐linear associations are hypothesized. To address these concerns and ensure a more equitable evaluation of the racial differences in health‐related parameters, independent of anthropometric influence, researchers have recommended matching Black and White participants based on key variables when conducting comparative analyses (Graybeal, Brandner, Aultman, et al. 2023; Graybeal, Tinsley, et al. 2023).

To that end, the purpose of this study was to prospectively identify VAT accumulation thresholds in a cohort of non‐Hispanic White and non‐Hispanic Black adults matched for sex, age, and body mass index (BMI) using a range of commonly employed whole‐body and abdominal‐specific adiposity measurements associated with chronic disease risk. We hypothesized that VAT thresholds would be uniquely identified for each race and race‐by‐sex subgroup, such that specific body composition or anthropometric predictors would reveal a threshold in one group but not in another. Furthermore, we hypothesized that when significant thresholds were present in both race groups, the thresholds would be significantly lower for White adults, particularly among White males.

## 2 Materials and Methods

This study was a secondary analysis derived from a larger body of research investigating the differences in physical and psychological health parameters between matched pairs of White and Black adults (Graybeal, Brandner, Aultman, et al. 2023; Graybeal, Tinsley, et al. 2023). As such, the recruiting and participant matching procedures detailed in the following sections have been thoroughly described elsewhere (Graybeal, Brandner, Aultman, et al. 2023; Graybeal, Tinsley, et al. 2023). However, these procedures are summarized for the present study, with additional information specific to the current analysis provided in greater detail. Notably, the data and analyses presented in this study are unique and serve as a supplementary extension of the broader research initiative.

### 2.1 Participants

A total of 366 non‐Hispanic White (100 F, 83 M) and non‐Hispanic Black (100 F, 83 M) males and females between the ages of 18–65 y were prospectively recruited for this cross‐sectional study from a rural region of the southern United States (Mississippi) due to resource availability. Participants were included if they were between the ages of 18–65 y and excluded if they were < 18 or > 65 y; missing any limbs or part of a limb; pregnant; trying to become pregnant; or lactating. Using the participant matching guidelines reported in prior investigations (Graybeal, Brandner, Aultman, et al. 2023; Graybeal, Tinsley, et al. 2023), Black participants who were eligible and completed the study were matched with a White participant based on their sex, age, and BMI. Among the 183 Black participants recruited, 10 (5 F, 5 M) had BMI values that could not be matched with a corresponding White participant. Additionally, 1 Black male participant had an age (64 y) that could not be matched with a White participant based on the guidelines put forth by the American Aging Association (Geifman et al. 2013; Graybeal et al. 2025).

Thus, 344 non‐Hispanic White (*n* = 172) and non‐Hispanic Black (*n* = 172) participants matched for sex (Female: 95 W, 95 B; Male: 77 W, 77 B), age (White: 23 ± 6 y; Black: 23 ± 6 y), and BMI (White: 26.7 ± 5.2 kg/m^2^; Black: 26.9 ± 5.6 kg/m^2^), were included in the final analysis. Participant characteristics are presented in Table 1. Importantly, there were no significant differences (all *p* ≥ 0.683) in age or BMI between White and Black adults (mean diff—age: −0.05y; BMI: −0.14 kg/m^2^), females (mean diff—age: 0.15y; BMI: −0.18 kg/m^2^), or males (mean diff—age: −0.29y; BMI: −0.09 kg/m^2^). This study was conducted according to the guidelines laid down in the Declaration of Helsinki and all procedures involving research study participants were approved by the university ethics committee (IRB#21‐213/23‐0446). Written informed consent was obtained from all participants prior to participation.

**TABLE 1:** Participant characteristics.

	Combined (*n* = 344)		Females (*n* = 190)		Males (*n* = 154)	
	White (*n* = 172)	Black (*n* = 172)	White (*n* = 95)	Black (*n* = 95)	White (*n* = 77)	Black (*n* = 77)
Weight Status (N/OW/O)	77/59/36	76/59/37	49/25/21	48/26/21	28/34/15	28/33/16
Abdominal Obesity	54 (31.4%)	44 (25.6%)	42 (44.2%)	34 (35.8%)	12 (15.6%)	10 (13.0%)
Visceral Obesity – Mass	85 (49.4%)	69 (40.1%)	47 (49.5%)	35 (36.8%)	38 (49.4%)	34 (44.2%)
Visceral Obesity – Ratio	56 (32.6%)	60 (34.9%)	8 (8.4%)	16 (16.8%)	48 (62.3%)	44 (57.1%)
Age (yrs)	22.5 ± 6.1	22.5 ± 6.1	22.8 ± 7.2	22.7 ± 7.2	22.0 ± 4.3	22.3 ± 4.4
Height (cm)	171.9 ± 9.8	171.6 ± 11.8	165.5 ± 6.4	163.7 ± 6.5	179.7 ± 7.3	181.3 ± 9.3
Weight (kg)	79.2 ± 17.6	79.5 ± 19.5	71.9 ± 14.9	71.1 ± 17.6	88.2 ± 16.5	89.9 ± 16.6
BMI (kg/m^2^)	26.7 ± 5.2	26.9 ± 5.6	26.3 ± 5.4	26.4 ± 5.8	27.3 ± 5.1	27.4 ± 5.4
Waist (cm)	89.6 ± 12.9	87.7 ± 13.9	88.2 ± 12.7	87.0 ± 14.8	91.4 ± 13.0	88.6 ± 12.8
Hip (cm)	103.2 ± 10.4	102.2 ± 10.5	102.9 ± 11.0	101.8 ± 11.1	103.6 ± 9.8	102.7 ± 9.8
WHR	0.87 ± 0.05	0.86 ± 0.06	0.86 ± 0.05	0.85 ± 0.07	0.88 ± 0.05	0.86 ± 0.06^a^
WHtR	0.52 ± 0.08	0.51 ± 0.09	0.53 ± 0.08	0.53 ± 0.09	0.51 ± 0.08	0.49 ± 0.08
Body Fat (%)	29.4 ± 10.8	27.4 ± 10.8	35.3 ± 8.1	33.2 ± 8.5	22.1 ± 9.0	20.3 ± 9.1
Fat Mass (kg)	23.6 ± 11.5	22.1 ± 12.0	26.2 ± 10.7	24.6 ± 11.7	20.5 ± 11.8	19.0 ± 11.7
Lean Soft Tissue (kg)	52.6 ± 12.9	54.0 ± 14.3	43.0 ± 5.9	43.6 ± 7.2	64.4 ± 8.6	66.9 ± 9.6
Trunk Body Fat (%)	30.9 ± 12.4	28.6 ± 12.6	36.1 ± 10.5	33.8 ± 11.0	24.6 ± 11.6	22.1 ± 11.4
Android Body Fat (%)	31.5 ± 14.8	28.9 ± 15.0	36.5 ± 13.3	34.1 ± 13.8	25.4 ± 14.2	22.4 ± 13.9
Gynoid Body Fat (%)	31.8 ± 11.7	30.4 ± 11.9	39.4 ± 7.4	37.7 ± 8.6	22.5 ± 8.9	21.5 ± 9.1
Android‐to‐Gynoid Body Fat %	0.97 ± 0.24	0.92 ± 0.23 ^a^	0.90 ± 0.21	0.88 ± 0.21	1.07 ± 0.24	0.97 ± 0.24^a^
VAT (g)	486.4 ± 465.0	397.1 ± 392.6	412.7 ± 410.6	345.5 ± 399.3	577.4 ± 512.6	460.8 ± 377.0
SAT (g)	1445.3 ± 1126.4	1223.5 ± 1131.4	1596.7 ± 1050.9	1380.3 ± 1095.4	1258.6 ± 1193.7	1030.0 ± 1152.2
VAT‐to‐SAT Ratio	0.56 ± 0.95	0.82 ± 1.5	0.24 ± 0.23	0.28 ± 0.40	0.97 ± 1.29	1.50 ± 1.98

*Note:* Data are presented as N (% of column total) or mean ± standard deviation.

Abbreviations: BMI: body mass index; N: normal weight; O: obese; OW: overweight; SAT: subcutaneous adipose tissue; VAT: visceral adipose tissue; WHR: waist‐to‐hip ratio; WHtR: waist‐to‐height ratio.

^a^ Significantly different from White participant counterpart.

### 2.2 Procedures

Given that DXA has shown to be robust to the lack of standardization (Tinsley et al. 2022), participants reported to the laboratory after an overnight fast from food, beverages, supplements and medication, and abstention from exercise for ≥ 8 h; similar to a typical overnight fast conducted in clinical settings. Participants were instructed to wear light “athletic” clothing without metal and any remaining external metal and/or accessories were removed before assessments were conducted. Participants then underwent several anthropometric assessments including height, weight, waist circumference (WC), hip circumference (HC), and body composition estimates collected by DXA (iDXA, General Electric, Boston, MA, USA).

### 2.3 Anthropometric Assessments and Dual‐Energy X‐Ray Absorptiometry

All anthropometric and DXA measurement procedures used in the current study have been described in detail elsewhere (Graybeal, Brandner, Tinsley, et al. 2023; Graybeal, Swafford, Compton, et al. 2024), but are summarized hereafter with additional information specific to this analysis described in greater detail. Upon arrival, participants had their height measured using a digital stadiometer and weight using a calibrated digital scale. WC and HC were measured at the level of the iliac crest and at the widest lateral portion of the hips, respectively, by trained investigators using a flexible non‐elastic aluminum tape measure, and the reliability of this protocol has been established in prior investigations (Graybeal, Brandner, Tinsley, et al. 2023). Waist‐to‐hip ratio (WHR) was calculated as WC divided by HC, and waist‐to‐height ratio (WHtR) was calculated as WC divided by height. Weight status was classified as normal weight (BMI < 25 kg/m^2^), overweight (BMI = 25–29.99 kg/m^2^), and obese (BMI ≥ 30 kg/m^2^), and abdominal obesity was defined as a WC ≥ 88 cm for females and ≥ 102 cm for males (Grundy et al. 2005).

Following the completion of all anthropometric measurements, body composition was assessed using a Lunar iDXA scanner equipped with enCORE software (v18), which provides validated estimates of android region fat, including VAT and SAT (Bennett et al. 2023; Kaul et al. 2012; Lee et al. 2020; Micklesfield et al. 2012). In addition to their well‐established validity, DXA‐derived VAT estimates have been shown to be more strongly associated with cardiometabolic health outcomes than MRI or CT (Bennett et al. 2023; Rothney et al. 2013; Schousboe et al. 2017). Participants were positioned in accordance with the manufacturer's guidelines. For participants whose body size exceeded the scanner's standard lateral dimensions, a reflection scanning method was employed, wherein the left side of the body (i.e., the left arm) was positioned outside the scanning field to allow full imaging of the right side. This technique has been shown to introduce minimal error when performed in larger participants (Moço et al. 2019; Tinsley et al. 2020). Android BF%, VAT mass (grams), and SAT mass (grams) were collected from the android region (abdominal), defined as the area between the iliac crest (inferior border of the measurement region) and 20% of the distance between the iliac crest and the chin (superior border of the measurement region) (Bosch et al. 2015; Kaul et al. 2012; Stults‐Kolehmainen et al. 2013). Gynoid BF% was collected from the gynoid region (hip/gluteal), defined as the area extending from the midpoint of the pelvis to the midpoint of the thigh (Bosch et al. 2015; Kaul et al. 2012; Stults‐Kolehmainen et al. 2013). The superior border (mid‐pelvis) was located below the iliac crest at a distance equal to 1.5× the height of the android region, while the inferior border (mid‐thigh) was defined as the point below the iliac crest located at twice the height of the android region. Android‐to‐gynoid BF% ratio was calculated as android BF% divided by gynoid BF%. Trunk BF% was collected and defined as the area between the iliac crest (inferior border) and the chin (superior border). BF%, fat mass, and lean soft tissue estimates were produced from whole‐body scans. To provide a normalized measure of adiposity across individuals with similar body sizes (i.e., BMI), relative rather than absolute adiposity metrics were used for analysis (i.e., BF% rather than fat mass). Relative visceral obesity was defined as having a VAT‐to‐SAT ratio of ≥ 0.40 (Emamat et al. 2024; Kaess et al. 2012), and absolute visceral obesity was determined as VAT ≥ 324 g for females and ≥ 391 g for males (Miazgowski et al. 2017).

### 2.4 Segmented Linear Regression Models

A total of nine anthropometric and body composition variables were included as independent predictor variables to identify VAT accumulation thresholds using segmented linear regression (SLR): BMI, WC, WHR, WHtR, whole‐body BF%, trunk BF%, android BF%, android‐to‐gynoid BF% ratio, and SAT. Specifically, SLR works by iteratively fitting segmental models, using the least squares approach, to identify a breakpoint (where the slope of the linear relationship changes), followed by the creation of separate regression models that assess the relationship of the data above and below the threshold (Bosch et al. 2015). Before identifying each threshold, SLR models require initialization, where the researcher provides an estimated breakpoint (typically based on common clinical cut‐off points; e.g., ≥ 30 kg/m^2^ as a clinical cutoff for obesity) to guide the algorithm in locating the actual change in slope (i.e., the breakpoint). As such, initialized values for BF% (M: 25%; F: 40%), WC (M: 102 cm; F: 88 cm), WHR (M: 0.90; F: 0.85), android BF% (M: 27%; F: 36%), trunk BF% (M: 20.5%; F: 23%), and android‐to‐gynoid BF% ratio (M: 1.0; F: 0.8) were based on existing sex‐specific clinical cut‐off points or the cut‐off points used in prior investigations (Bosch et al. 2015; Campa et al. 2025; Grundy et al. 2005; Minetto et al. 2021; Okosun et al. 2015). To contextualize the initialized SAT mass (M: 1800 g; F: 2600 g) and trunk BF% (M: 20.5%; F: 23%) values, we estimated the 75th and 50th percentile values, respectively, using a normal distribution model based on typical population means and standard deviations reported in large‐scale imaging studies (Lazarescu et al. 2025). Since the race‐specific analyses (i.e., White vs. Black adults) included both males and females, the averages of the male and female initialization values were used for this analysis. Because there are no sex‐specific clinical guidelines, initialized values of 30 kg/m^2^ for BMI and 0.50 for WHtR (Ashwell et al. 2012) were used in all SLR analyses.

### 2.5 Statistical Analysis

Participant characteristics (age, height, weight, BMI, etc.) were assessed between race (White vs. Black participants) and race within sex groups (e.g., White vs. Black females) using independent t‐tests (Table 1). Weight status (normal, overweight, obese), abdominal obesity, and absolute and relative visceral obesity prevalence between race and race within sex groups were assessed using chi‐squared tests of independence with continuity corrections for 2 × 2 tables (Graybeal et al. 2022; Graybeal, Brandner, Compton, et al. 2024). To verify the presence of associations between VAT (dependent variable) and each predictor variable, Pearson correlation coefficients and coefficients of determination (R^2^) were calculated for the overall sample, and by race and race within sex groups. The results of these initial associations are presented in (Supplemental Table 1). After significant associations were identified, SLR analyses were performed to determine a VAT accumulation threshold for each respective predictor variable.

Both race and race by sex specific thresholds were identified for each predictor variable. To determine if the coefficients (slopes) of the newly generated models were significant, linear regression analyses were conducted above and below the cut‐off points for each predictor variable. A breakpoint, identified by the SLR analysis, was considered to be significant if the relationship above, but not below this threshold, was statistically significant. Identified breakpoints were also considered to be statistically significant if they revealed two statistically significant but opposite directional relationships. For example, if the data below a given threshold demonstrated a significant negative relationship, and the data above the threshold demonstrated a significant positive relationship, then this was considered to be a significant change in slope for the relationship between VAT and the predictor variable in question. To further verify the existence of a breakpoint for VAT accumulation across predictor variables, we compared VAT above and below each identified threshold within and between race groups. To account for instances of unequal variance and large sample size discrepancies between groups, which could not be identified until SLR analyses (i.e., the study) were completed, a robust two‐way Welch's ANOVA with 20% trimmed means was used to evaluate the effects of race and threshold on VAT mass with Games‐Howell post hoc tests. Statistical significance was accepted at *p* ≤ 0.050. All statistical analyses were conducted using R.

## 3 Results

Overall, the sample was 55.2% female and 44.8% male (p_chi_ = 0.999). Between White and Black adults, there were no differences in the prevalence of weight classification (Combined: p_chi_ = 0.990; Males: p_chi_ = 0.977; Females: p_chi_ = 0.985), abdominal obesity (Combined: p_chi_ = 0.282; Males: p_chi_ = 0.818; Females: p_chi_ = 0.300), or absolute (Combined: p_chi_ = 0.104; Males: p_chi_ = 0.628; Females: p_chi_ = 0.107) and relative visceral obesity (Combined: p_chi_ = 0.732; Males: p_chi_ = 0.622; Females: p_chi_ = 0.126). Android‐to‐gynoid BF% ratio was significantly higher for White compared to Black participants in the combined sample (*p* = 0.033) and for males (*p* = 0.017). WHR was also significantly higher for White compared to Black males (*p* = 0.037). There were no significant differences between White and Black females for any variable (all *p* ≥ 0.052).

**FIGURE 1:**
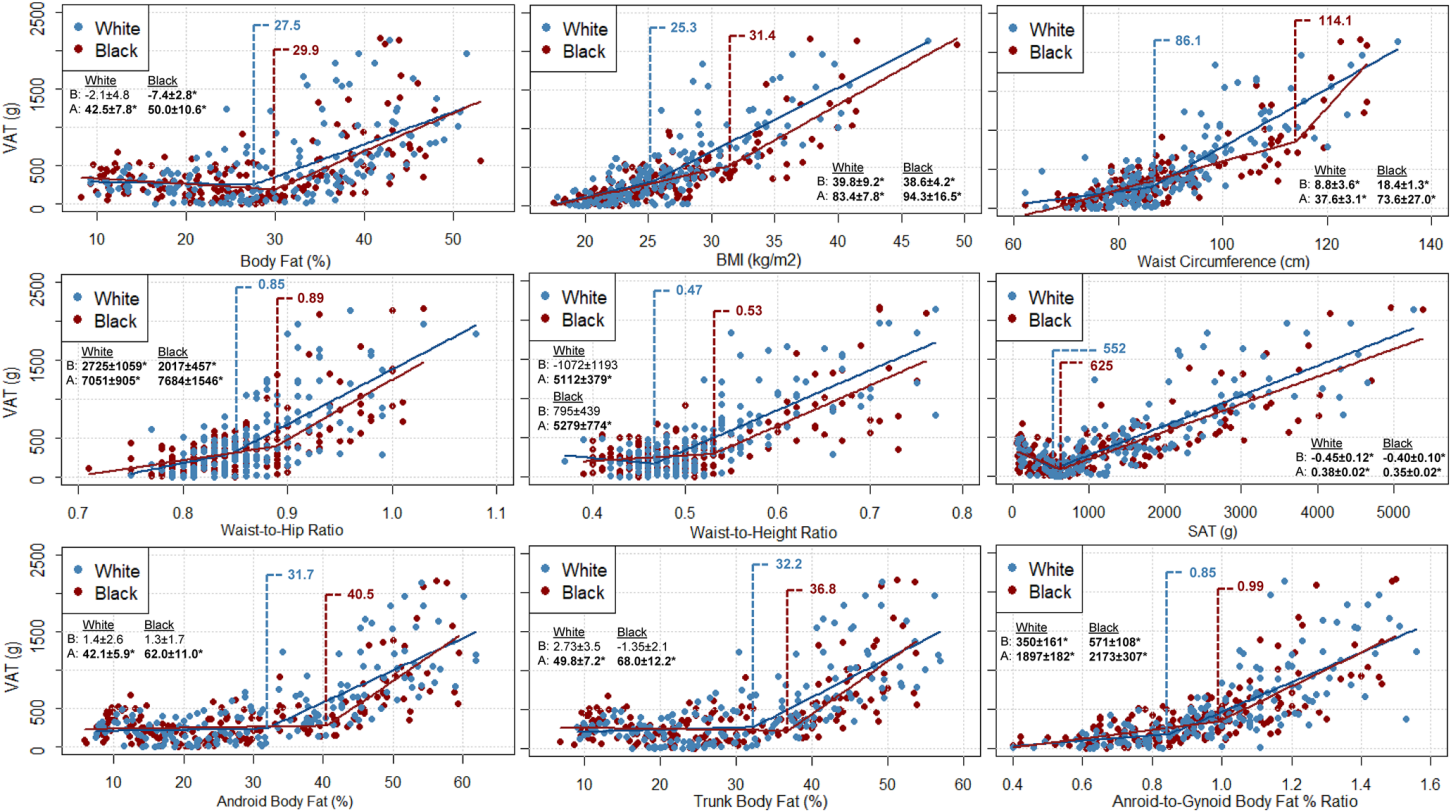
Scatterplots depicting segmented linear relationships between VAT mass and various anthropometric and body composition measures in Black (*n* = 172) and White (*n* = 172) adults. VAT mass is depicted on all y‐axes and body composition and anthropometric predictor variables are represented on each independent x‐axis. Blue and red markers and their corresponding regression lines represent the individual data points and segmented relationships between VAT and each predictor variable for White and Black participants, respectively. Blue and red vertical dashed lines represent the race‐specific VAT accumulation thresholds for White and Black participants, respectively. Slopes ± standard errors for the data above (A) and below (B) each race‐specific threshold are provided in each panel. *Slope statistically significant at *p* < 0.050. A: above; B: below; BMI; body mass index; VAT: visceral adipose tissue.

**FIGURE 2:**
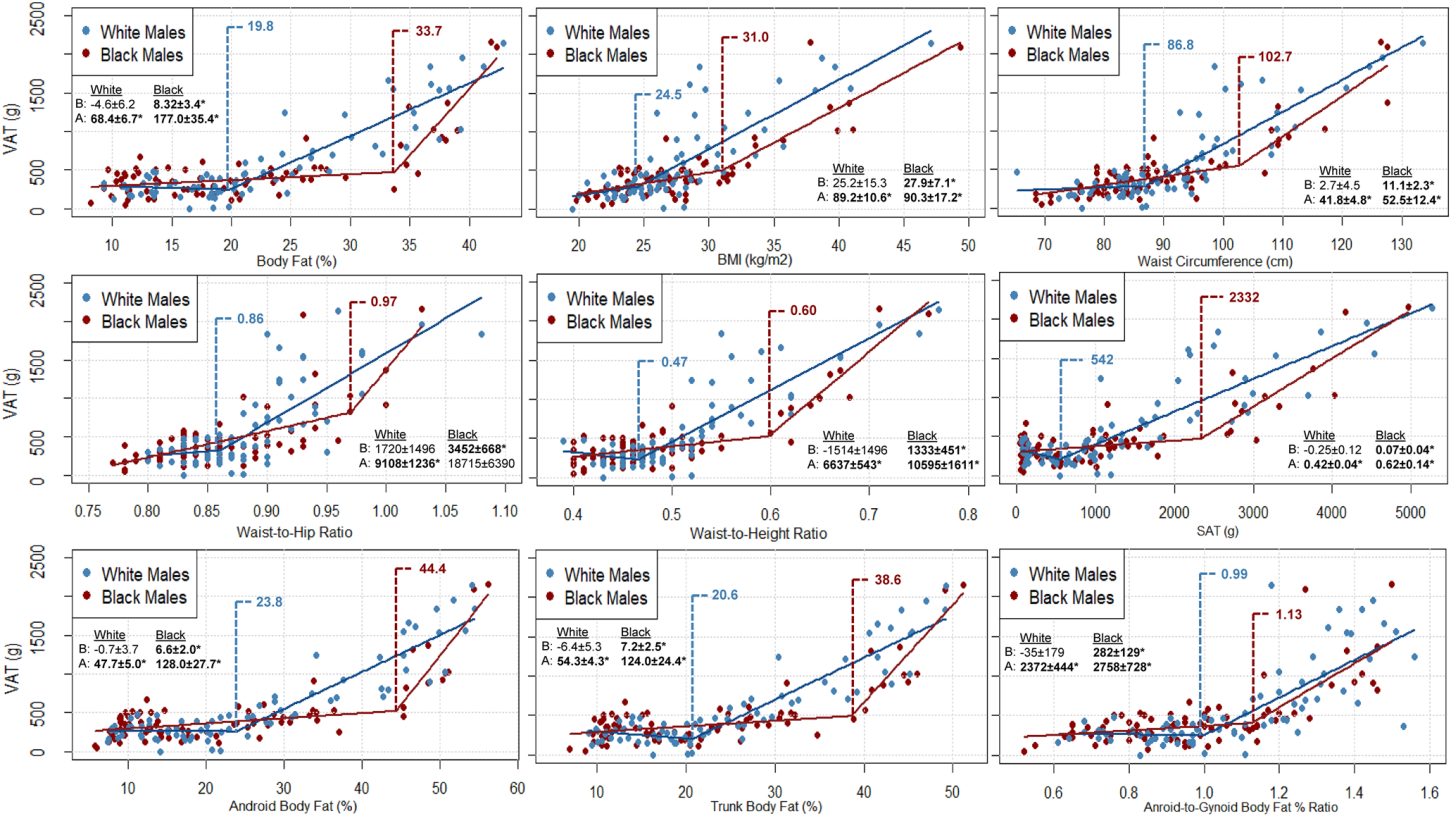
Scatterplots depicting segmented linear relationships between VAT mass and various anthropometric and body composition measures in Black (*n* = 77) and White (*n* = 77) males. VAT mass is depicted on all y‐axes and body composition and anthropometric predictor variables are represented on each independent x‐axis. Blue and red markers and their corresponding regression lines represent the individual data points and segmented relationships between VAT and each predictor variable for White and Black males, respectively. Blue and red vertical dashed lines represent the race‐specific VAT accumulation thresholds for White and Black males, respectively. Slopes ± standard errors for the data above (A) and below (B) each race‐specific threshold are provided in each panel. *Slope statistically significant at *p* < 0.050. A: above; B: below; BMI; body mass index; VAT: visceral adipose tissue.

**FIGURE 3:**
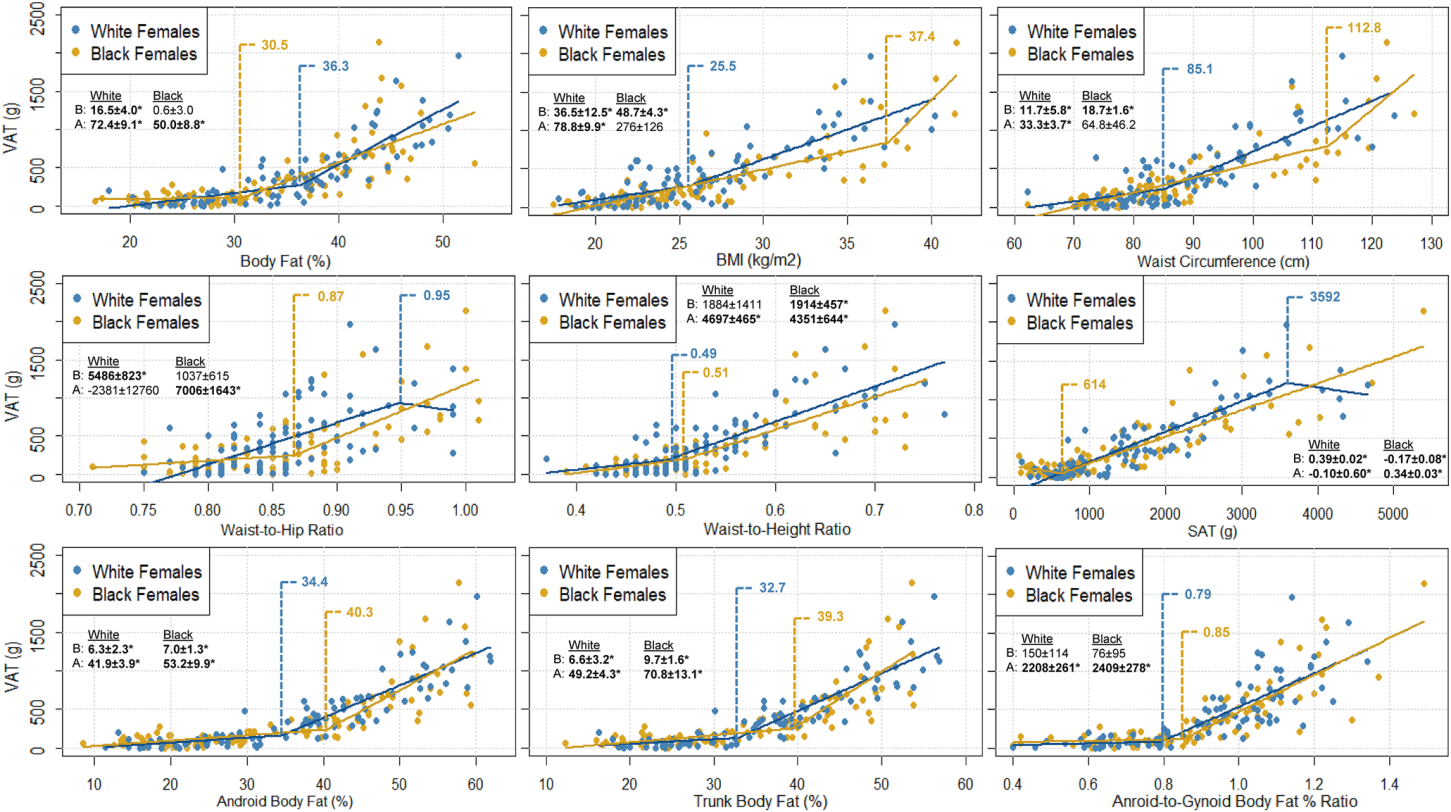
Scatterplots depicting segmented linear relationships between VAT mass and various anthropometric and body composition measures in Black (*n* = 95) and White (*n* = 95) females. VAT mass is depicted on all y‐axes and body composition and anthropometric predictor variables are represented on each independent x‐axis. Blue and red markers and their corresponding regression lines represent the individual data points and segmented relationships between VAT and each predictor variable for White and Black females, respectively. Blue and red vertical dashed lines represent the race‐specific VAT accumulation thresholds for White and Black females, respectively. Slopes ± standard errors for the data above (A) and below (B) each race‐specific threshold are provided in each panel. * Slope statistically significant at *p* < 0.050. A: above; B: below; BMI; body mass index; VAT: visceral adipose tissue.

Results of the SLR analyses identifying VAT thresholds and their corresponding coefficients above and below these thresholds—derived from simple linear regression—for Black and White adults, males, and females are illustrated in Figure 1, Figures 2 and 3, respectively. The results of the ANOVA examining the effect of VAT threshold on VAT mass are presented in Table 2, which compares values both within racial groups (e.g., VAT mass above vs. below each threshold among White participants) and between racial groups (e.g., VAT mass in White vs. Black participants below their respective thresholds). Table 3 presents these comparisons further stratified by sex within each racial group.

**TABLE 2:** VAT mass above and below identified thresholds in White and Black Adults.

	Threshold	Below Threshold		Above Threshold	
		*N*	VAT (g)	*N*	VAT (g)
Body Fat (%)	Mean ± SE		Mean ± SD		Mean ± SD
White	27.5 ± 2.5^†^	77	259 ± 217^a^	95	671 ± 528
Black	29.9 ± 1.7^†^	104	252 ± 182^a^	68	619 ± 510
BMI (kg/m^2^)					
White	25.3 ± 1.6	78	203 ± 159^a^	94	721 ± 503
Black	31.4 ± 1.4	139	265 ± 191^a^	33	952 ± 524
Waist (cm)					
White	86.1 ± 2.2	82	202 ± 165^a^, ^b^	90	746 ± 498^b^
Black	114.1 ± 2.2	161	328 ± 267^a^	11	1408 ± 542
WHR					
White	0.85 ± 0.02	68	221 ± 187^a^	104	660 ± 509
Black	0.89 ± 0.01	124	257 ± 185^a^	48	758 ± 535
WHtR					
White	0.47 ± 0.01^†^	38	189 ± 149^a^	134	571 ± 489
Black	0.53 ± 0.02^†^	120	243 ± 171^a^	52	753 ± 513
SAT (g)					
White	552 ± 112^†^	36	222 ± 133^a^	136	556 ± 496
Black	625 ± 89^†^	65	226 ± 162^a^	107	501 ± 452
Android Body Fat (%)					
White	31.7 ± 2.3^†^	93	214 ± 169^a^	79	807 ± 498
Black	40.5 ± 1.5^†^	124	242 ± 172^a^	48	797 ± 506
Trunk Body Fat (%)					
White	32.2 ± 2.0^†^	94	238 ± 210^a^	78	786 ± 510
Black	36.8 ± 1.3^†^	121	242 ± 174^a^	51	765 ± 508
Android‐to‐Gynoid Body Fat (%) Ratio					
White	0.85 ± 0.06	51	122 ± 117^a^	121	640 ± 471
Black	0.99 ± 0.04	105	218 ± 164^a^	67	678 ± 475

Abbreviations: BMI: body mass index; SAT: subcutaneous adipose tissue; SD: standard deviation; SE: standard error; VAT: visceral adipose tissue; WHR: waist‐to‐hip ratio; WHtR: waist‐to‐height ratio.

^†^ Represents a significant VAT accumulation threshold as determined by segmented linear regression analysis procedures.

^a^ Significantly different from above threshold within race group at *p* < 0.05.

^b^ Significantly different from Black adults within threshold group at *p* < 0.05.

**TABLE 3:** VAT mass above and below identified thresholds in White and Black Males and Females.

	Male					Female				
	Threshold	Below Threshold		Above Threshold		Threshold	Below Threshold		Above Threshold	
		*N*	VAT (g)	*N*	VAT (g)		*N*	VAT (g)	*N*	VAT (g)
Body Fat (%)	Breakpoint ± SE		Mean ± SD		Mean ± SD	Breakpoint ± SE		Mean ± SD		Mean ± SD
White	19.8 ± 1.3^†^	38	265 ± 116^a^	39	882 ± 565	36.3 ± 1.3	51	147 ± 153^a^	44	720 ± 401
Black	33.7 ± 0.74	65	348 ± 160^a^	12	1074 ± 589	30.5 ± 2.1^†^	40	93 ± 66^a^	55	530 ± 439
BMI (kg/m^2^)										
White	24.5 ± 1.9^†^	24	243 ± 110^a^	53	729 ± 551	25.5 ± 1.9	50	171 ± 174^a^	45	681 ± 432
Black	31.0 ±	62	340 ± 162^a^	15	960 ± 571	37.4 ± 1.2	90	291 ± 309^a^	5	1333 ± 574
Waist (cm)										
White	86.8 ± 2.5^†^	35	266 ± 123^a^	42	837 ± 568	85.1 ± 3.6	45	160 ± 183^a^	50	640 ± 426
Black	102.7 ± 2.7	67	349 ± 160^a^	10	1207 ± 548	112.8 ± 3.7	88	273 ± 284^a^	7	1262 ± 518
WHR										
White	0.86 ± 0.02^†^	28	284 ± 121^a^	49	745 ± 573	0.95 ± 0.05	89	383 ± 396	6	855 ± 397
Black	0.97 ± 0.01	72	405 ± 296	5	1259 ± 546	0.87 ± 0.02^†^	63	184 ± 168^a^	32	663 ± 520
WHtR										
White	0.47 ± 0.01^†^	22	262 ± 123^a^	55	704 ± 554	0.49 ± 0.03^†^	29	143 ± 184^a^	66	531 ± 427
Black	0.60 ± 0.01	66	346 ± 159^a^	11	1149 ± 554	0.51 ± 0.03	47	124 ± 98^a^	48	562 ± 461
SAT (g)										
White	542 ± 192^†^	26	283 ± 99^a^	51	727 ± 571	3592 ± 662	90	375 ± 385^a^, ^b^	5	1098 ± 223^a^, ^b^
Black	2332 ± 231	65	343 ± 159^a^	12	1098 ± 557	614 ± 168^†^	25	91 ± 67^a^	70	437 ± 429^a^
Android Body Fat (%)										
White	23.8 ± 1.9^†^	44	262 ± 119^a^	33	997 ± 535	34.4 ± 2.1	40	86 ± 91^a^	55	650 ± 388
Black	44.4 ± 1.0	66	346 ± 159^a^	11	1149 ± 554	40.3 ± 2.0	58	124 ± 93^a^	37	692 ± 448
Trunk Body Fat (%)										
White	20.6 ± 1.3^†^	36	253 ± 113^a^	41	862 ± 557	32.7 ± 1.6	38	83 ± 90^a^	57	633 ± 393
Black	38.6 ± 1.0	66	346 ± 159^a^	11	1149 ± 554	39.3 ± 1.5	60	133 ± 103^a^	35	710 ± 454
Android‐to‐Gynoid Body Fat (%) Ratio										
White	0.99 ± 0.06^†^	35	257 ± 107^a^	42	845 ± 563	0.79 ± 0.05^†^	29	62 ± 56^a^	66	567 ± 404
Black	1.13 ± 0.04	56	325 ± 145^a^	21	823 ± 542	0.85 ± 0.03^†^	42	95 ± 67^a^	53	544 ± 440

Abbreviations: BMI: body mass index; SAT: subcutaneous adipose tissue; SD: standard deviation; SE: standard error; VAT: visceral adipose tissue; WHR: waist‐to‐hip ratio; WHtR: waist‐to‐height ratio.

^†^ Represents a significant VAT accumulation threshold as determined by segmented linear regression analysis procedures.

^a^ Significantly different from above threshold within race group at *p* < 0.05.

^b^ Significantly different from Black counterpart within threshold group at *p* < 0.05.

### 3.1 Visceral Adipose Tissue Thresholds in the Combined Sample of White and Black Adults

All independent body composition and anthropometric variables revealed significant associations (all *p* < 0.001) with VAT in the combined (all *r* ≥ 0.51; R^2^ ≥ 0.26), White (all *r* ≥ 0.54; R^2^ ≥ 0.29), and Black (all *r* ≥ 0.47; R^2^ ≥ 0.22) participant samples (Supplemental Table 1). In summary, the identified VAT thresholds for all body composition and anthropometric variables were higher for Black compared to White adults. Significant VAT thresholds were observed for BF% (White: 27.5%; Black: 29.9%), WHtR (White: 0.47; Black: 0.53), SAT (White: 522 g; Black: 625 g), android BF% (White: 31.7%; Black: 40.5%), and trunk BF% (White: 32.2%; Black: 36.8%) in both White and Black adults (Figure 1). The significant thresholds identified for BF% and SAT in White and Black adults, as well as for WHtR in White adults, were below the clinical cut‐off points (BF%: 32.5%; SAT: 2200 g; WHtR: 0.50) used to initialize the model (see Segmented Linear Regression Models section).

For BF%, the relationship above (*p* < 0.001), but not below (*p* = 0.670) the threshold was significant for White participants. Additionally, a significant negative association below (slope: −7.4 ± 2.8 g; *p* = 0.010), and a significant positive association above (slope: 50.0 ± 10.6 g; *p* < 0.010) the BF% threshold was observed for Black participants. Similarly, significant negative associations were observed below (White slope: −0.45 ± 0.12 g; Black slope: −0.40 ± 0.10 g), and significant positive associations were observed above (White slope: 0.38 ± 0.02 g; Black slope: 0.35 ± 0.10 g) the SAT thresholds for both White and Black adults (all slopes *p* < 0.001). Significant relationships were observed above (all *p* < 0.001), but not below (all *p* ≥ 0.075) the identified thresholds for WHtR, android BF%, and trunk BF%.

The effect of threshold was significant for all body composition and anthropometric variables (all omnibus *p* < 0.001), with post hoc tests showing that VAT mass was significantly higher above compared to below each threshold for both White and Black participants (all *p* < 0.001; Table 2). The interaction between race and threshold was only significant for WC (omnibus *p* = 0.031), with post hoc tests revealing that Black participants had significantly higher VAT mass than White participants both above (*p* = 0.010) and below (*p* < 0.001) their respective WC thresholds. There were no other significant interactions between race and threshold (all omnibus *p* ≥ 0.092).

### 3.2 Visceral Adipose Tissue Thresholds in White and Black Males

All independent body composition and anthropometric variables revealed significant linear associations (all *p* < 0.001) with VAT in both White (all *r* ≥ 0.72; R^2^ ≥ 0.52) and Black males (all *r* ≥ 0.66; R^2^ ≥ 0.44) (Supplemental Table 1). The identified VAT thresholds for all body composition and anthropometric variables were higher in Black compared to White males (Figure 2). However, while significant thresholds were observed across all variables for White males (below threshold: all *p*  ≥ 0.050; above threshold: all *p* < 0.001), no significant thresholds were identified for Black males (below threshold: all *p* ≤ 0.046; above threshold: all *p* ≥ 0.061). All thresholds identified for White males (Table 3) were below the clinical cut‐off points (Clinical cutoffs—BF%: 25% BMI: 30 kg/m^2^; WC: 102 cm; WHR: 0.90; WHtR: 0.50; SAT: 1800 g; Android BF%: 27%; Android‐to‐gynoid BF%: 1.0) used to initialize the model with the exception of trunk BF% (White male threshold: 20.6%; Clinical cutoff: 20.5%).

In White males, the below‐threshold model for SAT approached significance (*p* = 0.050), but this relationship was negative (slope: −0.25 ± 0.12 g) and followed by a significant positive relationship above the SAT threshold (slope: 0.42 ± 0.04 g). Similarly, non‐significant negative associations were observed below the threshold for BF% (slope: −4.6 ± 6.2 g), WHtR (slope: −1514 ± 1496 g), android BF% (slope: −0.7 ± 3.7 g), trunk BF% (slope: −6.4 ± 5.3 g), and android‐to‐gynoid BF% ratio (slope: −35 ± 179 g) in White males (all *p* ≥ 0.230). The only non‐significant above‐threshold association was observed for WHR in Black males (*p* = 0.061), whereas all other above‐threshold models for both Black and White males were statistically significant (*p* ≤ 0.003).

The effect of threshold was significant across all body composition and anthropometric variables for both Black and White males (all omnibus *p* ≤ 0.002). Post hoc analyses revealed that VAT mass was significantly higher above compared to below each threshold for both groups (all *p* ≤ 0.004) with the exception of WHR for Black males, which did not reach statistical significance (Black: *p* = 0.079; White: *p* < 0.001). The interaction between race and threshold was only significant for WHtR (omnibus *p* = 0.015); however, post hoc tests did not reach statistical significance (both *p* ≥ 0.063). There were no other significant interactions between race and VAT threshold for White and Black males (all omnibus *p* ≥ 0.078).

### 3.3 Visceral Adipose Tissue Thresholds in White and Black Females

All independent body composition and anthropometric variables revealed significant linear associations (all *p* < 0.001) with VAT in both White (all *r* ≥ 0.60; R^2^ ≥ 0.36) and Black females (all *r* ≥ 0.68; R^2^ ≥ 0.46) (Supplemental Table 1). The identified VAT thresholds were higher in Black compared to White females for all body composition and anthropometric variables with the exception of BF%, WHR, and SAT (Figure 3; Table 3). Significant VAT thresholds were identified for BF% (Black females: 30.5%), WHR (Black females: 0.87), and SAT (Black females: 614 g) in Black females; for WHtR (White females: 0.49) in White females; and for android‐to‐gynoid BF% in both White and Black females (Black females: 0.79; White females: 0.85). All thresholds identified for White and Black females were below the clinical cut‐off points (Clinical cutoffs—BF%: 40% BMI: 30 kg/m^2^; WC: 88 cm; WHR: 0.85; WHtR: 0.50; SAT: 2600 g; Android BF%: 36%; Android‐to‐gynoid BF%: 0.8) with the exception of android‐to‐gynoid BF% in Black females (Black female threshold: 0.85).

For BF%, WHR, and android‐to‐gynoid BF%, significant associations were observed above the respective thresholds (all *p* < 0.001), but not below (all *p* ≥ 0.097), in Black females. Additionally, for the SAT threshold identified in this group, a significant negative association was found below the threshold (slope: −0.17 ± 0.08 g; *p* = 0.046), while a significant positive association was observed above the threshold (slope: 0.34 ± 0.03 g; *p* < 0.001). Among White females, significant relationships were observed above the identified thresholds for WHtR and android‐to‐gynoid BF% (both *p* < 0.001), but not below (all *p* ≥ 0.193). Additionally, non‐significant negative associations were observed above the thresholds for WHR (slope: −2381 ± 12 760 g) and SAT (slope: −0.10 ± 0.60 g) in White females (both *p* ≥ 0.861). Conversely, among Black females, the only non‐significant positive above‐threshold associations were observed for BMI and WC (both *p* ≥ 0.117), while all other above‐threshold models for both Black and White females demonstrated statistically significant positive associations (all *p* ≤ 0.001).

The effect of threshold was significant across all body composition and anthropometric variables for both Black and White females (all omnibus *p* ≤ 0.008). Post hoc analyses revealed that VAT mass was significantly higher above compared to below each threshold for both groups (all *p* ≤ 0.049) with the exception of WHR for White females, which did not reach statistical significance (Black: *p* < 0.001; White: *p* = 0.111). The interaction between race and threshold was significant for BF% (omnibus *p* = 0.012) and SAT (omnibus *p* < 0.001). Post hoc analyses revealed that SAT was significantly higher in White compared to Black females both above (*p* = 0.003) and below (*p* < 0.001) the thresholds. Post hoc tests for BF% did not reach statistical significance (both *p* ≥ 0.108). There were no other significant interactions between race and VAT threshold for White and Black females (all omnibus *p* ≥ 0.059).

## 4 Discussion

The purpose of this study was to prospectively identify VAT accumulation thresholds in a cohort of non‐Hispanic White and non‐Hispanic Black adults matched for sex, age, and BMI using a range of commonly employed whole‐body and abdominal‐specific adiposity measurements associated with chronic disease risk. Importantly, the goal of our study was not to reconfirm known racial differences in VAT levels, but rather to identify the inflection points at which VAT begins to accumulate more rapidly during BF gain, and to determine whether these thresholds differ between White and Black males and females; fostering a better understanding of the mechanisms driving these differences and promoting more equitable health outcomes.

A recent study evaluated VAT, body composition and anthropometric cutoffs in relation to disease risk (Potter and Friedl 2025). While both VAT and clinical adiposity measures (i.e., BF%, BMI, WC), which were significantly associated with VAT, showed good predictive performance for classifying cardiometabolic disease using sex‐intended cutoffs, relying on dichotomous classification systems for cardiometabolic disease presents well‐established diagnostic limitations (DeBoer and Gurka 2017; Gurka et al. 2014; Newsome et al. 2025). Our study is distinct in its focus on identifying thresholds for the onset of excess VAT accumulation, enabling earlier detection of disease risk rather than diagnosing existing disease, which may be too late for preventive intervention. Although prior studies have explored sex‐specific VAT cutoffs for body composition measures such as BF% (Bosch et al. 2015), these thresholds may not accurately reflect true VAT accumulation risk across racial groups. This is particularly relevant for Black adults, who tend to have lower VAT levels than White adults despite experiencing disproportionately higher rates of obesity, CVD, and T2D (Borkowski et al. 2024; Hales et al. 2020; Mensah 2018). While BF% has been shown to be a superior predictor of chronic disease risk compared to BMI (Mainous et al. 2025; Potter et al. 2025), it remains underutilized in routine practice, where simpler measures with established diagnostic cutoffs are still widely adopted. Moreover, while some studies have reported racial differences in specific body composition components (Kuchnia et al. 2017), others have found these differences to be negligible when controlling for relevant anthropometric variables using more sophisticated regression models (Barbosa‐Silva et al. 2005). However, studies that have matched Black and White participants on key physical attributes, similar to our study, have consistently found no significant differences in most body composition parameters, with the notable exception of VAT (Graybeal, Tinsley, et al. 2023; Kanaley et al. 2003).

To our knowledge, this is the first study to establish race‐specific VAT accumulation thresholds using both whole‐body and regional anthropometric measurements, as well as both clinical and research‐grade adiposity measurements, in a rigorously matched sample of Black and White adults. Overall, our findings not only demonstrate that VAT accumulation thresholds are statistically different across racial groups; the large differences observed for almost all thresholds between White and Black males and females highlight their clinical relevance and potential utility in improving risk assessment. Specifically, the same significant VAT thresholds were identified in the combined sample of Black and White adults (i.e., thresholds for BF%, WHtR, SAT, android BF%, trunk BF%), yet were consistently lower in White adults. When stratified by sex, significant VAT thresholds were observed across all variables for White males, whereas no significant thresholds were identified for Black males. Both Black and White females exhibited significant VAT thresholds, though with greater variability as the identified thresholds were unique to each group (Black female thresholds: BF%, WHR, SAT; White female thresholds: WHtR). VAT mass was also significantly higher above nearly all identified thresholds, supporting the existence of a physiological inflection point. Finally, most significant VAT thresholds fell below current clinical cutoffs, particularly among White males and Black females. Collectively, these findings demonstrate that VAT accumulation thresholds differ across racial groups, and that relying on universal cutoffs may be inadequate. Notably, the observation that many of the identified thresholds fall below the existing clinical guidelines highlights the need for more specific diagnostic criteria that account for racial and ethnic differences in fat accumulation patterns. Such refinements could enhance risk stratification, reduce disease misclassification, and improve the early detection of cardiometabolic conditions for populations at the greatest risk.

While prior studies have examined the influence of sex and race on VAT accumulation thresholds (Watson et al. 2019), methodological limitations, such as significant differences in critical anthropometrics (age, weight, fat mass, lean mass, bone mass, VAT mass) and imbalanced sample sizes that allow one group to span a broader range of adiposity phenotypes, warrant caution during interpretation. Thus, it is unsurprising that the BF% thresholds identified in our study (White male: 19.8%; Black female: 30.5%) were generally lower across all groups compared to prior reports (White male: 22.8%; Black female: 35.6%) (Watson et al. 2019). Because our matched‐pairs design enabled the use of a simplified model that minimizes the influence of typically uncontrolled anthropometrics, our finding that Black adults tend to accumulate VAT at higher levels of BF% and other abdominal‐specific measures, when compared to purposefully matched White adults, suggests that VAT accumulation is either delayed or less sensitive to increases in overall adiposity in Black adults; thereby providing strong evidence for race‐specific distribution patterns during the development of this pathogenic fat depot. Although these findings provide a clearer understanding of race‐specific fat distribution patterns, this is particularly concerning, as Black adults—especially Black males—demonstrate the greatest risk of developing cardiometabolic diseases, indicating that VAT alone may not fully explain disease risk in this population. This is supported by previous studies showing that after controlling for age and BMI, estimates of abdominal obesity, such as those used in the current study, are associated with metabolic syndrome estimates in young White adults, but not young Black adults (Graybeal, Compton, Swafford, et al. 2024). There are several potential explanations for this paradox, of which are discussed hereafter.

The differences in VAT accumulation we observed between Black and White adults may be attributable to racial variations in SAT distribution patterns. Black adults, particularly Black women, tend to store fat as SAT rather than the more metabolically harmful VAT (Katzmarzyk et al. 2010; Preis et al. 2010), which may explain why VAT levels are lower despite higher overall obesity rates. This is supported by our finding that Black, but not White females, demonstrated a significant negative association between VAT and SAT below the VAT threshold, suggesting preferential storage in SAT depots during the early stages of fat accumulation. Conversely, White females showed a significant positive linear relationship throughout (no significant threshold identified), indicating concurrent fat accumulation in both SAT and VAT compartments. As previously mentioned, when fat accumulation becomes excessive, it mechanically strains subcutaneous adipocytes and redirects storage to VAT (“i.e., the spillover effect”). Considering our findings, it is possible that Black adults demonstrate a greater ability to store fat in SAT depots longer before redirecting it to VAT due to differences in SAT expansion capacity, though the explanation for this remains ambiguous. Previous studies suggest that Black females have greater lipid storage capacity in gluteofemoral SAT depots (Rahman et al. 2009), supported by studies showing greater limb‐to‐trunk ratio (Wagner and Heyward 2000), and lower lipid turnover compared to White females, who exhibit higher lipolysis and de novo lipogenesis (White et al. 2018). This may lead to earlier saturation of SAT stores and redirection of excess lipids to visceral depots in White adults, supporting the notion that the higher VAT thresholds observed in Black compared to White adults may reflect enhanced lower‐body fat storage capacity and reduced lipid synthesis from nonlipid precursors in this group (White et al. 2018).

The earlier VAT thresholds observed in White adults may also contribute to a self‐reinforcing feedback loop between VAT accumulation and insulin resistance during periods of excess energy storage, potentially explaining the earlier and more rapid VAT accumulation in this group. Specifically, elevated VAT facilitates increased free fatty acid (FFA) release into portal blood, transporting excess lipids directly to the liver where they promote hepatic lipid synthesis (i.e., de novo lipogenesis) and impair insulin signaling (Klein 2004). VAT can also more effectively convert inactive cortisone to active cortisol via the enzyme 11β‐hydroxysteroid dehydrogenase (11β‐HSD1), which may further promote central fat accumulation and contribute to hepatic insulin resistance (Stimson et al. 2009). Notably, this effect may be amplified in White adults, who exhibit a greater cortisol response to stress compared to Black adults (Chong et al. 2008). As insulin resistance progresses, VAT becomes less responsive to insulin's antilipolytic effects, further increasing the release of FFA and glycerol, ultimately perpetuating hepatic lipid overload and progressive insulin resistance (Nielsen and Jensen 2024). Additionally, impaired hepatic 11β‐HSD1 activity at higher BF levels may lead to compensatory increases in cortisol production within VAT, ultimately amplifying local cortisol activity and further facilitating metabolic dysfunction (Stimson et al. 2009). VAT is also more prone to inflammation, which exacerbates insulin resistance and elevates circulating inflammatory markers (Verboven et al. 2018); effects that have been shown to be more pronounced in White compared to Black adults (Fisher et al. 2012; Hakim et al. 2020). Thus, if White adults experience earlier VAT accumulation, greater de novo lipogenesis, reduced subcutaneous fat storage capacity, heightened VAT inflammation, and a more reactive cortisol profile, this may initiate and sustain a metabolic feedback loop that accelerates VAT accumulation earlier and at lower BF levels relative to Black adults.

Regarding the sex‐ and race‐specific findings of our study, we observed that in White males, VAT accumulation followed a more predictable pattern across all body composition and anthropometric indicators. In contrast, Black males exhibited a less consistent relationship between body composition and VAT, with no identifiable breakpoint for rapid accumulation. This suggests a more linear relationship between VAT and adiposity in Black males. However, our findings also showed that White males had stronger positive linear associations between VAT and adiposity than Black males. These findings may indicate that standard clinical markers may be less effective in identifying VAT‐related health risks in Black males, potentially due to alternative physiological mechanisms that influence disease development. One possible explanation is that Black males tend to have different non‐fat tissue distribution patterns during weight gain compared to their White counterparts. Specifically, Black adults, particularly males, tend to gain more fat‐free mass relative to total weight gain compared to White males (Broyles et al. 2011). This may result in lower relative fat accumulation, less saturated SAT depots, and improved insulin sensitivity given the higher metabolic demands of fat‐free mass, all of which provide greater protection against rapid VAT accumulation. Another possible explanation could be that Black and White males demonstrate differences in sex hormone concentrations. Androgens and estrogens are implicated in depot‐specific adipose distribution, where elevated androgen levels are associated with increased VAT accumulation, while higher estrogen levels are associated with greater SAT distribution (Blouin et al. 2008). Additionally, sex‐hormone binding globulin (SHBG), a sex‐hormone transport protein, has been associated with protection against VAT accumulation (Azrad et al. 2012; Graybeal, Brandner, Wise, et al. 2024). Given that Black males tend to have similar androgen levels, but higher estradiol and SHBG concentrations compared to White males (Rohrmann et al. 2007), it is plausible that these hormonal differences promote a greater propensity for SAT accumulation which, in turn, may confer some protection against VAT accumulation.

Conversely, VAT accumulation patterns in females were less consistent, with some thresholds reaching significance in one racial group but not the other. Notably, significant VAT thresholds were identified for BF%, WHR, android‐to‐gynoid BF% ratio, and SAT (discussed above) in Black females. With the exception of the android‐to‐gynoid BF% ratio, these thresholds were lower than those observed in White females, suggesting that VAT accumulation may begin earlier in Black females for these adiposity measures. While this pattern implies that the higher thresholds observed in the combined sample were likely driven by the elevated, but non‐significant, thresholds observed in Black males, it is important to note that no significant thresholds were found for these variables in White females, aside from the android‐to‐gynoid BF% ratio; suggesting that White females may exhibit a more linear relationship between overall adiposity and VAT accumulation (i.e., where both increase simultaneously). Notably, the only significant threshold (android‐to‐gynoid BF% ratio) shared between these groups was higher for Black than White females suggesting that when significant thresholds are identified across groups, they tend to be higher in Black than in White females. As observed in males, it is possible that these findings are due to sex hormone differences between White and Black females. Specifically, Black females tend to have similar estradiol levels, but higher SHBG and lower androgen concentrations compared to White females (Sutton‐Tyrrell et al. 2005). Although different from the hormonal profile observed in Black males, this hormonal profile may similarly contribute to a protective effect against VAT accumulation in this group. Importantly, we observed a significant WHtR threshold in White females that warrants further discussion. Prior studies have shown that weight and fat mass scale significantly with height in White, but not Black females (Heymsfield et al. 2014). However, because WC and VAT tend to scale less linearly with height (Brown et al. 2022), and because White females were non‐significantly taller than Black females in our sample, it is possible that the modest differences between groups reflect a slight overestimation of VAT in shorter White females. Therefore, had these scaling biases not existed, this relationship may have been more linear. Despite this, WHtR has previously demonstrated the strongest association with metabolic syndrome severity among White females when compared to other clinical measures of abdominal obesity (Graybeal, Compton, Swafford, et al. 2024). Regarding the significant thresholds observed for the android‐to‐gynoid BF% ratio in both groups, it appears that, regardless of race, there is a point at which SAT depots in the gluteofemoral region become saturated and direct fat to VAT compartments. Although this threshold was lower in White females, this ratio may still provide valuable insight into VAT‐related health risk in females overall, and should be explored further in future research.

Our finding that many of the observed VAT thresholds—particularly in White males and Black females (White male BF% threshold: 19.8%; Black female BF% threshold: 30.5%)—fell below current clinical cutoffs (Males: 25%; Females: 40%) and those reported in prior studies (White males and all males: ≥ 22.8%; Black females and all females: ≥ 35.6%) (Bosch et al. 2015; Watson et al. 2019) carries important clinical implications. Specifically, this suggests that VAT accumulation may begin at lower levels of body fat or anthropometric indices than current guidelines recognize. As a result, individuals may be classified as “low risk” based on traditional thresholds, despite already having clinically meaningful levels of VAT and associated cardiometabolic health risks. This misclassification could delay diagnosis or intervention, allowing disease progression to go unnoticed. This concern is especially relevant for Black females, who are at an elevated risk for conditions such as CVD and T2D, yet may be overlooked due to reliance on conventional cutoffs that do not adequately reflect their risk profile. As such, updating VAT thresholds to account for race‐specific patterns could enhance the accuracy of cardiometabolic risk assessment. However, given that disease risk in Black adults is not fully captured by body composition metrics alone, clinicians should avoid overreliance on superficial physical attributes when evaluating health status in this population (Graybeal, Compton, Swafford, et al. 2024). Instead, comprehensive assessments that consider these VAT thresholds should serve as tools to promote health equity by preventing under‐ and misdiagnosis in a group that has historically faced disproportionate health burdens. By contextualizing VAT thresholds within broader social and environmental factors, and applying them in a race‐aware, but not race‐based manner, practitioners can align culturally appropriate treatment approaches with the ethical principles of equity and justice, ensuring that these thresholds are used to reduce disparities rather than perpetuate them. Awareness of the potential for perpetuating stereotypes is a particularly important consideration for clinicians, as studies have shown that social factors such as discrimination have a similar impact on VAT accumulation in Black and White adults (Lewis et al. 2011), and that discrimination is positively associated with increased WC in White, but not Black females (Hicken et al. 2018).

Several limitations of this study warrant further discussion. First, our analysis was limited to non‐Hispanic White and non‐Hispanic Black adults, which prevented the examination of VAT thresholds and distribution patterns in other racial and ethnic groups. However, to maintain a focused aim and address disparities in a population with disproportionately high cardiometabolic risk, we chose to examine Black adults exclusively. Future studies should apply similar methodological approaches to explore VAT thresholds across a broader range of racial and ethnic populations. While our sample was not exclusively composed of young adults, the average age of our sample was relatively low. Notably, 97.4% of participants were between 18 and 39 years old—a group shown to experience the most rapid increases in cardiometabolic risk (Hirode and Wong 2020)—highlighting the need for early disease detection strategies in this age range. Nevertheless, future research should assess whether the observed VAT thresholds and distribution patterns are consistent across older age groups. Although the validity of DXA‐derived VAT estimates is well established and shows strong correspondence with cardiometabolic health outcomes comparable to MRI and CT, differences in VAT measurement locations exist between DXA models (e.g., Hologic vs. General Electric). Therefore, our findings should be interpreted specifically within the context of General Electric systems, and researchers and clinicians using other models are advised to apply correction factors, where available, before drawing conclusions from VAT data (Bennett et al. 2023). Our study sample was relatively small. However, our rigorous matching procedures minimized the influence of key anthropometric differences and sample size imbalances that have confounded prior studies. Additionally, all matched participants resided in the same US region during the study period. While this may limit generalizability compared to larger national datasets, it allowed us to control for regional lifestyle factors, such as diet and physical activity, that could otherwise influence fat distribution patterns. Using BMI to match our participants, as opposed to other measurements of body size or adiposity, is also a limitation of our study. However, the global burden of disease risk is still attributable to BMI (Zhou et al. 2024), and BMI remains the primary estimate of body size in clinical practice. Furthermore, there were no differences in BF%, WC, fat mass, lean mass, height, or weight between groups despite matching participants by BMI. Additional socioeconomic factors were not controlled for during matching or within our analyses. However, this was intentional, and we have previously described the biases that result from excessive control and model overfitting (see Introduction section). Finally, we did not collect measurements of genetic ancestry and instead relied on self‐identified race as a proxy. However, neither race nor genetic ancestry are manipulable variables, which limits their utility in causal inference for health outcomes in human models. Since both are observational and neither can definitively predict individual VAT distribution patterns, both race and genetic ancestry (along with their associated biological underpinnings) may serve as proxies for exposure rather than determinants.

## 5 Conclusions

In conclusion, our study demonstrates that, after controlling for key anthropometric variables through rigorous matching procedures, distinct VAT accumulation thresholds exist between Black and White adults across a range of clinical and research‐grade adiposity measurement techniques. Notably, these thresholds were most consistently observed in White males and Black females, with few identified in White females and none in Black males. Furthermore, the finding that VAT accumulation begins at lower levels of adiposity than current clinical guidelines suggest, particularly in White males and Black females, emphasizes the limitations of universal cutoffs in accurately identifying early cardiometabolic risk. Together, the presence of sex‐ and race‐specific VAT thresholds, often below established clinical standards, supports the need for population‐specific screening tools to enhance early detection and intervention strategies. However, the absence of significant thresholds in Black males and the variability observed in females suggest that VAT alone may not fully capture disease risk in Black adults, reinforcing the importance of comprehensive, multifactorial assessments in these populations. Future research should extend these analyses to include broader racial and ethnic groups, a wider age range, and diverse body composition phenotypes to better understand VAT accumulation patterns and their implications for cardiometabolic health.

## Author Contributions

All authors contributed to the study conception and design and have approved the final manuscript. Conceptualization, data curation, formal analysis, investigation, methodology, project administration, supervision, resources, and roles/writing – original draft were performed by Austin J. Graybeal. Conceptualization, formal analysis, and visualization were performed by Nuno Oliveira. Data curation, investigation, methodology, validation, and writing – review and editing were performed by Molly F. Johnson, Maria G. Kaylor, Abby T. Compton, Sydney H. Swafford, and Caleb F. Brandner. Conceptualization, methodology, resources, and writing – review and editing was performed by Jon Stavres.

## Conflicts of Interest

The authors declare no conflicts of interest.

## Supporting information


**Supplemental Table 1.** Associations between body composition parameters and visceral adipose tissue mass.

## Data Availability

The data that support the findings of this study are available from the corresponding author upon reasonable request.
