# Supplementary material for: Clinical Thresholds for Visceral Adiposity Accumulation: A Comparative Analysis in Sex‐, Age‐, and BMI‐Matched Black and White Adults
Source: Am J Hum Biol. 2025 Nov 5;37(11):e70165. doi: 10.1002/ajhb.70165 (PMC12587260; doi:10.1002/ajhb.70165)
Supplement: Supplementary file 1 — Supplemental Table 1. Associations between body composition parameters and visceral adipose tissue mass. [file AJHB-37-e70165-s001.pdf]

Supplemental Table 1. Associations between body composition parameters and visceral adipose tissue mass [a]

	Combined (n = 344)			Females (n = 190)		Males (n = 154)	
	**Total (n = 344)**	**White (n = 172)**	**Black (n = 172)**	**White (n = 95)**	**Black (n = 95)**	**White (n = 77)**	**Black (n = 77)**
BMI (kg/m^2)	.81 (.65)	.81 (.66)	.82 (.68)	.83 (69)	.82 (.67)	.80 (.64)	.82 (.68)
Waist (cm)	.82 (.68)	.82 (.68)	.83 (.69)	.82 (.68)	.84 (.70)	.83 (.69)	.83 (.69)
WHR	.68 (.46)	.69 (.47)	.68 (.47)	.60 (.36)	.68 (.46)	.76 (.58)	.69 (.48)
WHtR	.75 (.56)	.77 (.59)	.73 (.54)	.80 (.64)	.79 (.63)	.85 (.73)	.81 (.65)
Body Fat (%)	.51 (.26)	.54 (.30)	.47 (.22)	.82 (.68)	.70 (.49)	.85 (.72)	.69 (.48)
Trunk Body Fat (%)	.64 (.40)	.67 (.46)	.59 (.35)	.86 (.75)	.77 (.59)	.87 (.76)	.73 (.53)
Android Body Fat (%)	.68 (.46)	.72 (.52)	.62 (.39)	.86 (.74)	.77 (.59)	.87 (.76)	.73 (.53)
Android-to-Gynoid Body Fat %	.74 (.55)	.75 (.57)	.72 (.52)	.78 (.61)	.77 (.59)	.72 (.52)	.66 (.43)
SAT (g)	.79 (.63)	.81 (.65)	.78 (.61)	.87 (.75)	.85 (.31)	.85 (.72)	.79 (.62)

Data are presented as r (R^2). [a] all associations with visceral adipose tissue mass (g) statistically significant at $p < 0.001$
